# Study on Fiber-Fabric Hierarchical Reinforcement for High-Toughness Magnesium Phosphate Cement Composites

**DOI:** 10.3390/polym17212844

**Published:** 2025-10-24

**Authors:** Weipeng Feng, Yuan Fang, Chengman Wang, Peng Cui, Kunde Zhuang, Wenyang Zhang, Zhijun Dong

**Affiliations:** 1 Institute of Technology for Future Industry, School of Science and Technology Instrument Application Engineering, Shenzhen University of Information Technology, Shenzhen 518172, China; fengwp@sziit.edu.com; 2 Guangdong Provincial Key Laboratory of Durability for Marine Civil Engineering, College of Civil and Transportation Engineering, Shenzhen University, Shenzhen 518060, China; yuanfang@szu.edu.cn (Y.F.); wangchm@szu.edu.cn (C.W.); 2150471024@email.szu.edu.cn (P.C.); 2015090154@email.szu.edu.cn (K.Z.); wyzhangluck@163.com (W.Z.); 3 Guangdong Engineering and Technology Research Center for Low-Carbon and Energy-Saving Building, Shenzhen 518060, China

**Keywords:** magnesium phosphate cement, hybrid fiber reinforcement, carbon fiber-reinforced polymer, toughness

## Abstract

Magnesium phosphate cement (MPC) has gained attention in specialized construction applications due to its rapid setting and high early strength, though its inherent brittleness limits structural performance. This study developed an innovative toughening strategy through synergistic reinforcement using hybrid fibers and carbon fiber-reinforced polymer (CFRP) fabric capable of multi-scale crack control. The experimental program systematically evaluated the hybrid fiber system, dosage, and CFRP positioning effects through mechanical testing of 7-day cured specimens. The results indicated that 3.5% fiber dosage optimized flexural–compressive balance (45% flexural gain with <20% compressive reduction), while CFRP integration at 19 mm displacement enhanced flexural capacity via multi-scale reinforcement. Fracture analysis revealed that the combined system increases post-cracking strength by 60% through coordinated crack bridging at micro (fiber) and macro (CFRP) scales. These findings elucidated the mechanisms by which fiber–CFRP interaction mitigates MPC’s brittleness through hierarchical crack control while maintaining its rapid hardening advantages. The study established quantitative design guidelines, showing the fiber composition of CF/WSF/CPS15 = 1/1/1 with 19 mm CFRP placement achieves optimal toughness–flexural balance (*f*_f_/*f*_c_ > 0.38). The developed composite system reduced brittleness through effective crack suppression across scales, confirming its capability to transform fracture behavior from brittle to quasi-ductile. This work advances MPC’s engineering applicability by resolving its mechanical limitations through rationally designed composite systems, with particular relevance to rapid repair scenarios requiring both early strength and damage tolerance, expanding its potential in specialized construction where conventional cement proves inadequate.

## 1. Introduction

Magnesium phosphate cement (MPC) is a chemically bonded ceramic material characterized by rapid setting and high early strength [1]. The typical MPC system comprises magnesium oxide (MgO) as the base component, an acidic phosphate solution (e.g., NH_4_H_2_PO_4_ or KH_2_PO_4_) as the reactant, and borax as a retarder [2]. The chemical interaction between these constituents results in the formation of crystalline magnesium phosphate phases, primarily struvite or k-struvite, endowing the final product with significant sustainability advantages, including CO_2_ sequestration, utilization of industrial wastes in production, and applications in hazardous waste stabilization and rapid low-energy repairs [3,4,5,6,7]. Given the growing emphasis on sustainable construction, MPC has emerged as a versatile candidate with applications spanning structural rehabilitation, biomedical engineering implementations, and hazardous waste containment systems.

Despite the above merits, MPC exhibits inherent limitations in flexural performance, characterized by higher brittleness and lower toughness compared to conventional cement-based materials [8,9,10]. The pronounced mechanical vulnerability of MPC stems from its crystalline-dominated microstructure, where rapid formation of dense struvite crystals (MgNH_4_PO_4_·6H_2_O) creates preferential cleavage planes with minimal crack deflection capacity [11], unlike Portland cement’s amorphous phases and fiber-like hydration products, such as calcium silicate hydrate (C-S-H) gels, which contribute to energy dissipation and crack bridging in cementitious systems [12,13,14]. Given this inherent brittleness, research efforts have focused on fiber reinforcement strategies to reconcile MPC’s high compressive strength with enhanced flexural toughness required for structural applications [11,14,15,16,17]. Fiber reinforcements such as steel fiber, polyvinyl alcohol (PVA) fiber, polypropylene (PP) fiber, basalt fiber, etc., were incorporated into MPC matrices to enhance ductility [5]. The underlying toughening mechanism involves both physical and chemical interactions: at the microscale, fibers bridge microcracks through interfacial bonding with the crystalline struvite matrix, thereby delaying crack coalescence and growth [18,19,20]. It is confirmed by Dong et al. that aligned fibers exert closure stress across crack planes through frictional pull-out, inhibiting crack widening while dissipating fracture energy [21]. At the nanoscale, chemical bonding between fiber surfaces and MPC matrices has been demonstrated through phosphate-mediated interactions (e.g., P-O-Mg bonds) [22]. This dual-scale synergy transforms brittle fracture into a multi-stage failure process: Initial microcracking activates fiber bridging, followed by debonding and fiber pull-out, which collectively enhance energy absorption by up to 400% [23]. Consequently, the resulting fiber-reinforced MPC composites demonstrate improved energy absorption capacity and overall toughness while maintaining the original advantages of rapid setting and high early strength [24,25].

**Figure 1:**
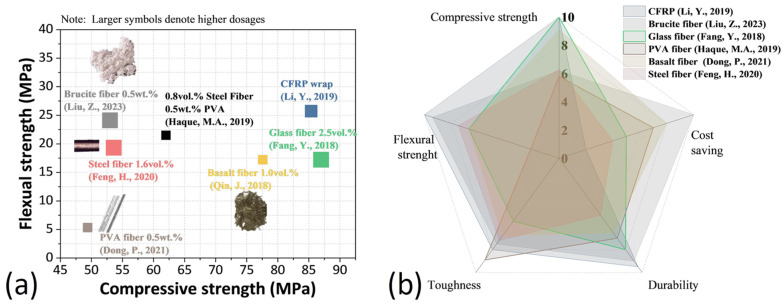
Comparison of the effects of different fiber types and CFRP on MPC performance, summarized from [5,11,14,24,25,28,33]: (**a**) Correlation between compressive strength and flexural strength; (**b**) Normalized results evaluated against five key parameters (note: durability and cost savings were explicitly mentioned or derivable in the cited studies).

Figure 1 summarizes the comparison results of the effects of different fiber types and CFRP on MPC performance. Haque et al. reported that the inclusion of steel fiber, PVA fiber, and basalt fiber with dosages ranging from 0.6% to 1.2% increased the flexural strength of MPC by over 15%, though with slight compressive strength reduction [26]. E-glass and polyester fibers, which can be destroyed by cement reaction corrosion, were also found to be compatible with MPC [27]. With the investigation of the interface transition zone (ITZ) between MPC and steel fiber, Feng et al. found that the bonding performance between steel fiber and MPC was better than that between steel fiber and sulphoaluminate or ordinary Portland cement [28]. Due to the excellent bonding performance, Zhu et al. [29] and Feng et al. [30] found that steel fibers can increase the flexural strength and toughness of MPC concrete and mortar with unchanged or slightly improved compressive strength. Fang et al. [24] and Tassew and Lubell [31] found a pronounced effect of glass fiber on increasing the flexural strength of MPC mortar and concrete, but had little influence on their compressive strengths and moduli of elasticity. Qin et al. [14] noticed better MPC reinforcement by basalt fiber over glass fiber, and the flexural strength and fracture toughness of basalt fiber-reinforced MPC increased with the fiber dosage. However, these fibers may not be suitable for the long-term service of MPC owing to the proneness of steel fibers to corrosive degradation and the inferior alkaline durability of glass and basalt fibers [11]. By contrast, organic compound fibers, such as PVA fiber, were relatively stable in alkali or corrosive media [14], making them an excellent choice for environments where corrosion of steel reinforcement could be a concern, such as marine applications. Dong et al. [25] found that PVA fibers with a dosage of up to 0.75% significantly improved fracture toughness and ductility performance and increased the porosity of the reinforced MPC mortar. The poor interfacial bonding between organic fibers and cementitious materials is still an issue for fiber-reinforced MPC products [11]. Therefore, to achieve the MPC-based composite with high strength and ductility, adding different types of fibers into the MPC mixture was applied, as using hybrid fibers to reinforce cementitious materials is beneficial for compensating for the shortcomings of single fiber additions [32].

While fiber reinforcement effectively addresses MPC’s brittleness limitations, advanced composite materials offer additional enhancement pathways. Carbon fiber reinforced polymer (CFRP) is a lightweight, strong material that can reinforce concrete columns through MPC bonding. Previous research shows that using CFRP-MPC composites can strengthen reinforced concrete construction [33]. According to Li et al. [33], with the MPC-bonded CFRP composites, the compressive strength of the concrete column was increased by 85.39%. Ding et al. [34] and Li et al. [35] found excellent interface bonding performances of the MPC with various CFRPs. These advantages make CFRP a suitable material for enhancing MPC materials.

Building upon the established benefits of both fiber reinforcement and CFRP-MPC systems, an emerging opportunity lies in the synergistic combination of hybrid fibers with CFRP fabrics to optimize the mechanical performance of MPC. While existing studies have demonstrated the effectiveness of CFRP in enhancing compressive strength [27] and excellent interfacial bonding with MPC [34,35], the potential of integrating these two reinforcement approaches remains largely unexplored. This integration adheres to the fundamental principle of hybrid reinforcement in cementitious composites, wherein distinct reinforcement mechanisms operate synergistically to achieve performance characteristics beyond the capabilities of individual reinforcement systems [33]. Therefore, the complementary interaction of these systems establishes a hierarchical reinforcement architecture capable of mitigating the inherent limitations of each method when applied in isolation.

The primary objective of this study is to investigate the synergistic toughening effects of hybrid fiber and CFRP fabric reinforcement in MPC composites, with a comprehensive evaluation of their combined influence on mechanical performance and durability characteristics. This study systematically investigated the effects of fiber type and CFRP fabric placement configuration on the mechanical properties of MPC mortar, with a particular focus on compressive strength and flexural performance. The optimal design was obtained by achieving the maximum flexural to compressive strength value. Given the primary research objective of toughness enhancement, a comprehensive analysis of flexural force-deflection responses was conducted to quantify key performance indicators, including toughness indices, energy absorption capacity, etc., providing fundamental insights into the synergistic reinforcement mechanisms of the hybrid fiber–CFRP system in MPC mortar. This study holds significant potential to advance sustainable construction materials by synergistically combining the rapid-setting and eco-friendly attributes of MPC with the mechanical reinforcement and durability enhancements provided by hybrid fibers and CFRP fabric. The resulting composite system offers a promising solution for developing resilient, sustainable, and cost-effective infrastructure materials capable of meeting modern engineering demands while reducing environmental impact.

## 2. Materials and Methods

### 2.1. Materials

The raw materials used for MPC production consisted of dead-burned magnesia (DBM: MgO, 98% purity), mono-potassium phosphate (MPP: KH_2_PO_4_, 98% purity), borax (BR: Na_2_B_4_O_7_·10H_2_O, industrial grade), quartz sand (QS), and tap water (W). The DBM was purchased as fine powders with an average particle diameter of 45 μm. Before mixing the raw materials, MPP and BR were milled using a high-speed pulverize and sieved through a 1 mm sieve to enhance their reactivity during the subsequent mixing process.

Different fiber types, along with carbon fiber-reinforced polymer (CFRP) fabric, were used to create a hybrid reinforcement system for the prepared MPC.

Table 1 presents the dimensions and colors of the fibers used. The glass fiber powder (GFP) was produced by milling the glass fiber (GF), and the maximum particle size of the GFP was 150 μm.

### 2.2. Experimental Design and Sample Preparation

**Figure 2:**
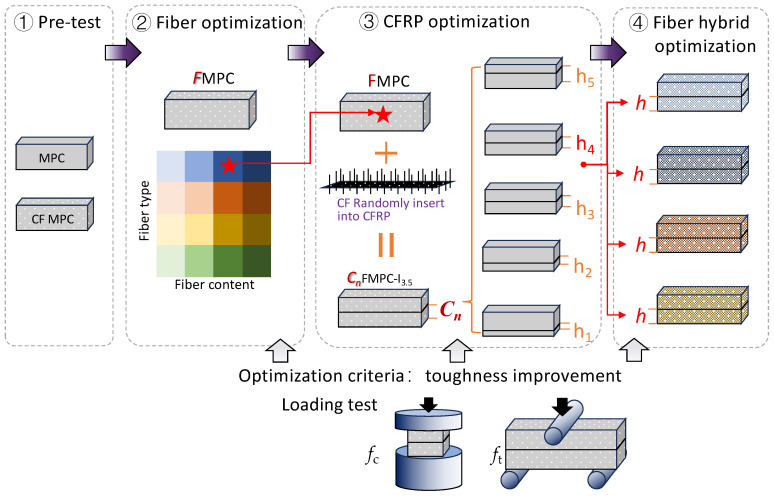
Schematic diagram of experimental procedures: the optimal design was obtained by the *f*_f_/*f*_c_ and toughness index via compressive and flexural loading tests.

A schematic diagram shown in Figure 2 presents the overall experimental design of this study. The whole investigation is divided into five steps, including pre-tests, optimal fiber MPC selection, CFRP addition, optimal CFRP location determination for toughness improvement, and verification of the cooperative toughness-improving effect of other fibers and CFRP. Among the above steps, compressive and flexural loading test results were used to evaluate the toughness-improving ability of different mix designs. The details of these loading tests and the calculation of relevant parameters are described in Section 2.3 and Section 2.4.

The experimental procedures is schematically illustrated in Figure 2. In the pre-tests (Figure 2①), the mix design for MPC (Table 2) was optimized and the reinforcing effect of CF incorporation was verified. To enhance the toughening effect of MPC, hybrid fiber systems were developed by incorporating supplementary fibers with CF, resulting in a series of fiber-reinforced MPC composites. In the second experimental phase (Figure 2②), systematic compression and flexural testing were conducted on specimens incorporating varying fiber types and contents to identify the optimal fiber reinforcement configuration for MPC composites. After that, the CFRP fabric with CF fibers randomly inserted (Figure 2③) was added to the optimal fiber-reinforced specimen during the molding period. It is noted that about 1/3 length of the CF was inserted into the CFRP fabric to form a cactus-like surface before the mixing. The CFRP fabrics were strategically positioned at predetermined vertical locations within the specimen geometry, with their elevations precisely corresponding to the interface of the initially cast cementitious matrix layer. The CFRP fabric was placed on top of the first layer of the pouring paste after vibration, and the remaining pastes were poured onto the CFRP fabric until the mold was filled. To account for potential CFRP flotation during placement, a positional tolerance of ±1 mm was permitted. After determining the optimal CFRP place with the loading tests, this assembly and molding method was subsequently applied to other fiber MPC designs to verify the efficacy of this cooperative toughness-improving effect.

Table 2 lists the mixed designs of all the specimens used in this study. The specimens were prepared with the dimensions of 40 mm × 40 mm × 40 mm for compressive strength (*f*_c_) tests and 40 mm × 40 mm × 160 mm for flexural strength (*f*_f_) tests. Following casting, the specimens were subjected to 2 min of vibration for compaction, then cured under controlled conditions at 20 ± 2 °C and 60 ± 5% relative humidity. The specimen labeling system follows this convention: *C_n_*FMPC-*T_m_*, where
MPC:Magnesium phosphate cement.F:Fibers.*C_n_*:CFRP fabric placed at *n* mm (9, 13, 16, and 19; CFRP placed in Table 2).*T_m_*:Fiber type designation (I, II, III, and V1-V5, Fiber type in Table 2).*m*:Fiber content in weight percentage (*m* = 1.5, 2.5, 3.5, and 5, Fiber column in Table 2).

### 2.3. Mechanical Property Tests

Considering that MPC attains ca. 90% of its 28-day compressive strength within 7 days of curing, all mechanical characterization in this study was performed on specimens cured for 7 days. The compressive and flexural strength tests were performed according to the procedure recommended by the Chinese National Standard GB/T 17671-2021 [36] using a universal testing machine (Shenzhen Enpuda Industrial System Co., Ltd., Shenzhen, China). Cubic specimens with dimensions of 40 mm × 40 mm × 40 mm were subjected to uniaxial compression testing at a controlled loading rate of 2.4 kN/s. The compressive strength (*f*_c_) was determined following Equation (1). Flexural strength (*f*_f_) evaluation was performed using a three-point bending configuration (Figure 2) on beam specimens, with displacement control at 0.5 mm/min, and the resulting values were calculated using Equation (2).

(1) $$f_{c}=\frac{F_{max}}{A}$$

(2) $$f_{f}=\frac{1.5PL}{{bd}^{2}}$$

where *F_max_* is the maximum load at failure, N; A the area under compression, mm^2^; *P* is the ultimate load at failure, N; L is the span between the two bottom supports Figure 2②), equal to 100 mm in this study; b is the width of the beam specimens, mm; d is the height of the beam specimens, mm.

The coefficient of variation (COV) of three parallel tests was computed to evaluate the dispersion of the strength results obtained. The COV values were obtained according to Equation (3) below.

(3) $$\mathrm{COV}\;(\%)=\frac{Standard\;deviation\;(\sigma )}{Mean\;(\mu )}\times 100$$

The change rate (R) of the mechanical properties due to the fiber and fabric reinforcement was obtained by Equation (4).

(4) $$R\;(\%)=\frac{f_{test}-f_{control}}{f_{control}}\times 100$$

### 2.4. Parameters of Flexural Properties

**Figure 3:**
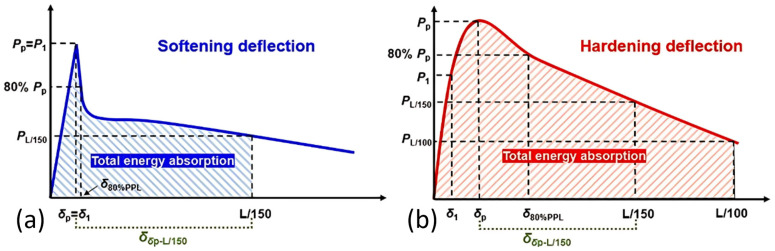
Typical load-deflection (L-D) curves of fiber-reinforced cementitious materials: (**a**) softening deflection and (**b**) hardening deflection characteristics.

Figure 3 shows typical load-deflection (L-D) curves of fiber-reinforced cementitious materials, which can be classified as softening behavior and hardening behavior. Basic flexural parameters are illustrated in Figure 3, with the corresponding names and definitions listed in Table 3. The first peak load (*P*_1_) is considered the load causing the first cracking in a sample, with the load increasing linearly with deflection to reach this peak. In softening deflection curves, the first peak load (*P*_1_) equals the peak load (*P*_p_) (Figure 3a), while in hardening deflection curves, the peak load (*P*_p_) is greater than the first peak load (*P*_1_) (Figure 3b). In addition to the basic flexural parameters, samples’ properties, such as post-peak behavior, toughness, energy absorption (EA), and stiffness, can also be obtained by calculating with the information extracted from the L-D curves. More details regarding the calculation of these flexural properties are described in Table 3.

## 3. Results and Discussion

### 3.1. Effect of Fiber Types and Dosages

#### 3.1.1. Mechanical Strengths

**Figure 4:**
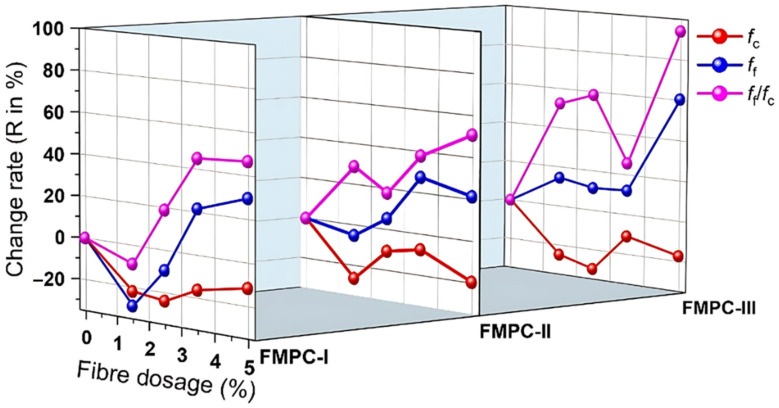
Change rates of *f*_c_, *f*_f_, and *f*_f_/*f*_c_ of FMPCs with fiber dosages ranging from 0 to 5%.

As detailed in Table 4, the compressive strength (*f*_c_), flexural strength (*f*_f_), and their corresponding ratio (*f*_f_/*f*_c_) of fiber-reinforced MPC composites are presented for different fiber types and dosages. The initial reduction in compressive strength (*f*_c_), particularly at lower fiber dosages like 1.5%, primarily stems from the introduction of air voids during fiber mixing and potential localized stress concentrations at the fiber–matrix interface, which acts as flaws under compressive loading [37]. Conversely, the increase in flexural strength (*f*_f_) is attributed to the fibers’ ability to bridge microcracks that develop under tensile bending stresses. This bridging effect transfers load across cracks, delaying their propagation and requiring more energy to cause failure [38], thereby enhancing *f*_f_ and material ductility. The results are compared to the unreinforced control MPC via change rates (R). The addition of fibers induced a marked reduction in *f*_c_ but a pronounced enhancement in *f*_f_, yielding elevated *f*_f_/*f*_c_ ratios. This behavior indicates a preferential improvement in ductility and fracture toughness over compressive strength, which yielding elevated prior observations by Feng et al. [31]. The COV for *f*_c_ is relatively low for all the tested specimens compared to the COV for *f*_f_. This suggests that flexural strength measurements are more sensitive to factors such as specimen preparation, fiber distribution, and loading conditions. The higher variability in *f*_f_ relates directly to the critical dependence of flexural performance on fiber dispersion and alignment within the brittle MPC matrix [25]. Imperfect fiber distribution can lead to localized weak planes or variations in fiber density across the tension zone during bending, significantly influencing the measured strength [39]. To better evaluate the changes in mechanical strengths due to fiber dosages, the fiber-dosage-dependent change rates (R) of *f*_c_, *f*_f_, and *f*_f_/*f*_c_ are further plotted in Figure 4.

For all FMPC types, the change rates (R) of *f*_f_/*f*_c_ are over those of *f*_c_ and *f*_f_ in all tested dosages, indicating that the tested fibers enhance the material’s ductility and toughness more than its compressive capacity. As the fiber dosages increased from 0 to 1.5%, all the FMPC samples exhibited a significant *f*_c_ reduction, and further increasing the dosage led to fewer negative R values. At 3.5% and 5% dosages, all the FMPC samples showed the R values of *f*_c_ higher than −20%. Flexural strength tended to improve with the fiber addition across all FMPC types, particularly at higher dosages, such as 3.5% and 5%. The R values of *f*_f_/*f*_c_ consistently increase with the fiber dosage for the FMPC samples, indicating a strong positive impact of fibers on the *f*_f_/*f*_c_ ratio. Specific to the type of fiber used and the overall mechanical performance with fiber dosages, FMPC-I and FMPC-II showed excellent performance at 3.5% and 5%, while FMPC-III displayed considerable performance at 5%.

#### 3.1.2. L-D Curves

**Figure 5:**
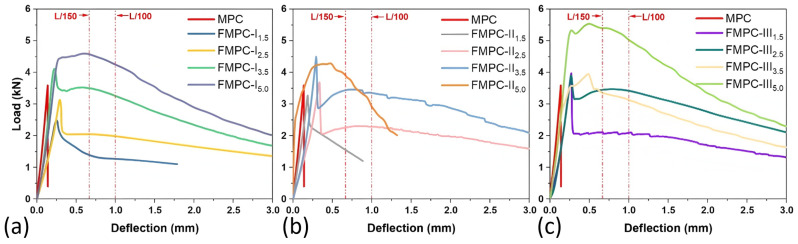
Flexural load-deflection (L-D) curves of MPC reinforced with various types and dosages of fibers: (**a**) MPC-I; (**b**) MPC-II; (**c**) MPC-III.

Figure 5 displays the load-deflection (L-D) curves, which present the flexural performance of MPC reinforced with different types and dosages of fibers. Initially, each curve ascends nearly linearly, reflecting a proportional increase in load with deflection, indicative of the material’s stiffness. This initial stiffness of the MPC was reduced by introducing fibers, as evidenced by a gentle softening of the slope. Upon reaching the peak load, the curves exhibit the composites’ maximum load capacity, corresponding to the ultimate flexural strength before the onset of significant deformation. For all the types of FMPC investigated here, the incorporation of fibers impacted the flexural characteristics substantially. As the fiber dosages increased from 1.5 to 5.0, increases in peak load capacity were noticed, suggesting the fibers’ effect in enhancing the flexural strength. The improved ductility was also noted with the fiber enhancement, as the reinforced MPCs can endure more extensive deflection before yielding to failure.

#### 3.1.3. Flexural Strengths, Moduli, and Deflections

**Figure 6:**
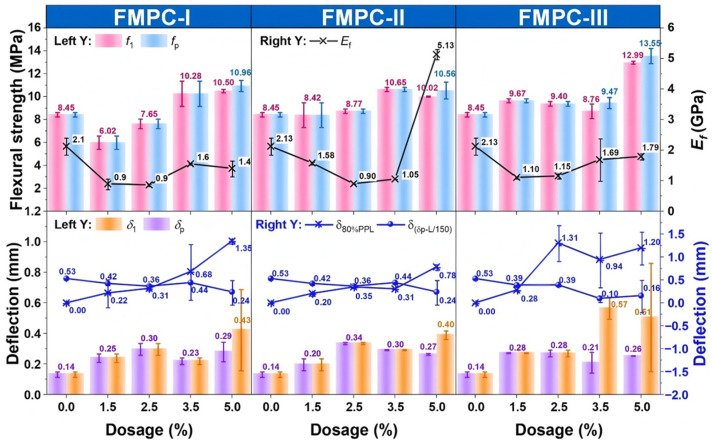
Strengths (MPa), moduli (GPa), and deflections (mm) of MPC reinforced with various types and dosages of fibers (Note: Error bars represent standard deviation (SD)).

To further analyze the flexural property changes with various types and dosages of fibers added in MPC, the results of the strength (*f*_1_) and corresponding deflection (*δ*_1_) at the first peak point, the strength (*f*_p_) and deflection (*δ*_p_) at the peak point, the modulus of elasticity (*E_f_*), the deflection (*δ*_80%PPL_) at 80% post-peak load, and the difference between the peak deflection (*δ*_p_) and the deflection of L/150 were obtained from the L-D curves in Figure 5, and depicted in Figure 6.

The data in Figure 6 reveals that, across all types of FMPC, up to a fiber dosage of 3.5%, the initial *f*_1_ is equal to the *f*_p_. This equivalence originates from insufficient fiber content to substantially modify matrix-dominated brittle fracture behavior, where initial microcracks rapidly propagate to failure without significant fiber-mediated load redistribution [40]. The *f*_1_ and *f*_p_ of the samples exhibit a general increase as the fiber dosage rose, regardless of the fiber types, attributed to progressive enhancement in crack-bridging efficiency as more fibers intercept propagating cracks and redistribute stresses through interfacial bond development [41]. Optimal flexural strengths were achieved at a 5.0% fiber dosage for FMPC-I and FMPC-III, while FMPC-II plateaus at 3.5%. Adding more fibers did not significantly affect the *f*_1_ and *f*_p_ of FMPC-II. In contrast to the strengths, fiber addition generally decreased the *E_f_*, implying a reduction in stiffness, except for FMPC-II at a 5.0% fiber dosage. The prevalent *E_f_* reduction stems from localized microcracking at fiber–matrix interfaces that disrupts elastic load transfer [42], while FMPC-II’s anomalous increase at 5.0% suggests densified fiber packing may momentarily enhance composite rigidity through confinement effects [43]. The largest *E_f_* values across the FMPC samples were observed at a 3.5% fiber dosage for FMPC-I and 5.0% for FMPC-II and FMPC-III, corresponding to region-specific optimization points where fiber content improves particle packing density without excessive interfacial defect generation [44].

The measured deflection parameters, including initial cracking deflection (*δ*_1_), peak load deflection (*δ*_p_), and post-peak deflection at 80% residual strength (*δ*_80%PPL_), exhibited a consistent positive correlation with increasing fiber content. This systematic enhancement in deformation capacity provides clear evidence of improved composite ductility. The observed behavior stems from intensified fiber–matrix interaction mechanisms, where higher fiber dosages promote more extensive fiber pull-out processes, thereby increasing energy absorption capacity and effectively postponing structural failure through delayed load-bearing framework disintegration [45]. Consequently, the differential between peak deflection and the deflection at the serviceability limit *δ*_(δp-L/150)_ decreased as fiber dosage increased. Despite the similar overall trend, the specific patterns of deflection change for each FMPC type are different. FMPC-I exhibits a consistent rise in deflection, whereas FMPC-II levels off beyond a 2.5% dosage, and FMPC-III shows significant fluctuations. Meanwhile, the increase in deflection variability at higher dosages, especially for FMPC-III, could indicate potential performance issues or measurement inconsistencies, possibly due to fiber aggregation at high dosages [46,47]. Caution is therefore advised when considering high-fiber dosage designs for FMPC-III, as the observed deflection irregularities may compromise the reliability and integrity of structures.

#### 3.1.4. Other Flexural Parameters

**Figure 7:**
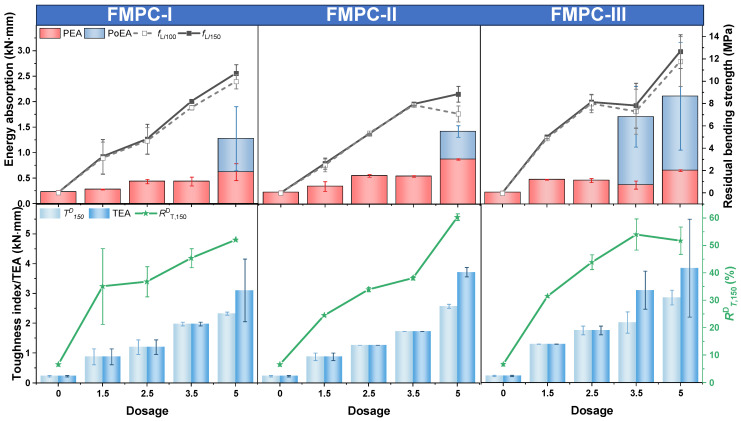
Energy absorption (kN·mm), residual bending strengths (MPa), toughness index (kN·mm), and *R*^D^_T,150_ of MPC reinforced with various types and dosages of fibers. (Note: Error bars represent standard deviation (SD)).

Figure 7 illustrates the influence of incremental fiber dosage augmentation on the energy absorption capacity, residual flexural strength, and fracture toughness characteristics across different fiber-modified MPC (FMPC) composites. For all the tested FMPCs, both the PEA and PoEA trended to ascend with increased fiber dosage, signifying that higher fiber content enhanced energy absorption capabilities and the overall toughness of the composites, consistent with study of [48]. Notably, a significant surge in PoEA was observed between the 3.5% to 5.0% dosage range, indicating a marked improvement in the material’s ability to absorb energy beyond the elastic stage. Concurrently, data variability also escalated with fiber dosage, pointing to increased experimental uncertainty at higher fiber contents.

As fibers play more significant roles after the occurrence of crack formation by acting as bridges to hinder the extension of flexural tensile cracks [49], using residual bending strengths (*f*_L/150_ and *f*_L/100_) can be a way to evaluate the impact of fibers on flexural strengths. Regarding the *f*_L/150_ and *f*_L/100_, FMPC-I showed a nearly linear improvement with rising fiber dosages. FMPC-II followed a similar upward trajectory up to a 3.5% dosage, beyond which the rate of increase for *f*_L/150_ tapered off and *f*_L/100_ slightly diminished. For FMPC-III, a leveling off in residual strengths occurred between 2.5% and 3.5% dosages, after which a significant data variability suggested potential reliability issues in the FMPC composites of high fiber content.

The toughness index (*T*^D^_150_), total energy absorption (TEA), and equivalent flexural strength ratio (*R*^D^_T,150_) exhibited consistent growth with increased fiber dosage across all the FMPC samples, improving the toughness of the composites. However, the pronounced error bars at higher dosages, particularly for FMPC-III, may reflect inconsistencies in fiber distribution or variability in matrix-fiber bonding. It underscored that while toughness tended to improve with fiber addition, the degree of enhancement may be variable and influenced by factors like fiber dispersion within the matrix.

#### 3.1.5. Selection of FMPC for CFRP Fabric Addition

Mechanical testing results indicate that fiber dosage has a more pronounced impact on the properties of FMPC samples than fiber type. A 5.0% dosage notably enhanced flexural properties and toughness and reduced compressive strength by no more than 25%. Yet, at this dosage, data variability also significantly increased, primarily due to fiber agglomeration and inhomogeneous dispersion during mixing [50,51]. The issue of poor repeatability stems from uneven fiber distribution creating areas of both high fiber concentration and insufficient reinforcement, which compromises load transfer mechanisms [52]. Studies on hybrid fiber-reinforced materials highlight this sensitivity to fiber volume fraction, where even low concentrations require uniform dispersion for consistent performance, while fiber-reinforced polymer systems similarly depend on well-dispersed fibers for mechanical integrity [53]. This challenge parallels findings in broader cement-based materials research, where material homogeneity proves critical for predictable behavior [54,55], including advanced systems like 3D-printable ceramics requiring precise dispersion control [56]. At high dosages, these effects intensify without specialized mixing techniques or dispersing agents [57]. Therefore, despite the improvements at 5.0% fiber content, such fundamental variability in fiber distribution and unstable fiber–matrix bonding renders this dosage problematic for practical applications.

For further exploration with subsequent CFRP fabric reinforcement, the 5.0% dosage was dismissed owing to its high variability. A 3.5% dosage was preferred, considering the observed toughness improvements. FMPC-I_3.5_ and FMPC-II_3.5_ emerged as superior options over FMPC-III_3.5_, which showed high data variability in multiple parameters. With comparable strength and flexural toughness, FMPC-I_3.5_ was chosen for its higher modulus of elasticity (*E_f_*), positioning it as the preferred substrate for CFRP fabric enhancement.

### 3.2. Effect of the Displacement of CFRP Fabric

Table 5 and Table 6 presents a comprehensive summary of mechanical properties including *f*_c_, *f*_f_ and *E*_f_, toughness index (TEA and *T*^D^_150_), TF and TWF across CFRP placements at 9, 13, 16, and 19 mm displacements, with subsequent subsections detailing the specific effects of CFRP positioning on MPC performance.

#### 3.2.1. Selection of FMPC for CFRP Fabric Addition

**Figure 8:**
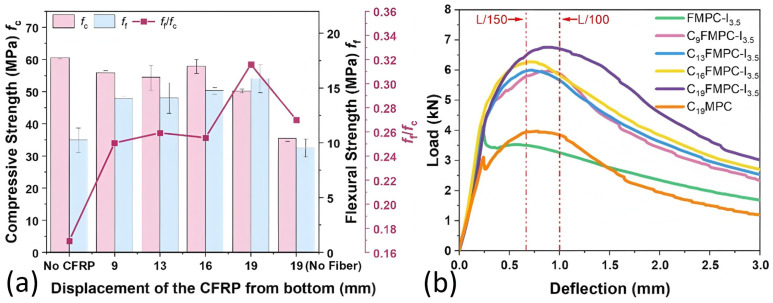
Properties (**a**) mechanical strengths (MPa) and (**b**) L-D curves of optimal fiber MPC reinforced with CFRP fabric. (Note: Error bars represent standard deviation (SD)).

Figure 8 delineates the mechanical strengths and flexural L-D curves of FMPC with variations in CFRP fabric displacements. Figure 8a demonstrates that incorporating CFRP fabric typically lowers the *f*_c_ but enhances the *f*_f_ of the FMPCs. A notable trend is observed where the *f*_c_ decreased and the *f*_f_ increased as the CFRP is positioned further from the base, peaking at 19 mm displacement for the highest *f*_f_/*f*_c_ ratio, indicating an optimal CFRP placement.

In the L-D curves of Figure 8b, all CFRP-reinforced FMPC specimens exhibit a consistent pattern: a vague yield point and an initial linear response followed by a gentle decline post-peak load, suggesting increased ductility. The FMPC curves without CFRP exhibit a steeper post-peak fall-off, while the CFRP-reinforced non-fiber MPC shows a distinct yield point. Overall, CFRP fabric reinforcement not only boosts load capacity but also augments the ductility of FMPC, allowing for greater deflection prior to failure.

#### 3.2.2. Flexural Strengths, Moduli, and Deflection

**Figure 9:**
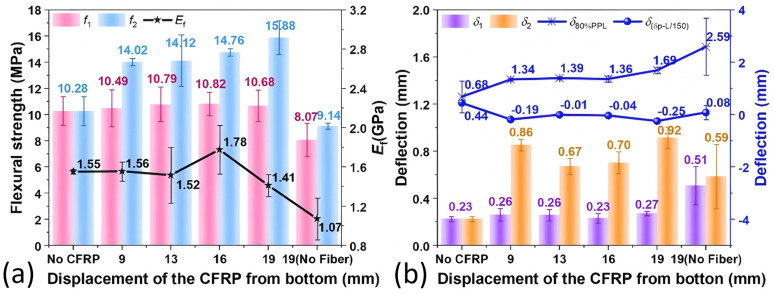
Flexural properties (**a**) strengths (MPa) and moduli (GPa), and (**b**) deflections (mm) of optimal fiber MPC reinforced with CFRP fabric. (Note: Error bars represent standard deviation (SD)).

Figure 9 demonstrates the effect of CFRP positioning variations on the flexural performance characteristics of FMPCs, including flexural strength, elastic modulus, and deformation behavior. Figure 9a shows that CFRP enhanced the peak flexural strength *f*_p_ more substantially than the initial strength *f*_1_, with *f*_p_ rising as CFRP was positioned further from the base. The modulus of elasticity (*E*_f_) exhibits limited variation with CFRP incorporation, attaining a peak value at 16 mm displacement. However, this observed maximum demonstrates significant statistical uncertainty, as evidenced by the substantial error margins. MPC without fibers registers the lowest flexural strength and modulus of elasticity, underscoring the reinforcement effect of fibers.

Figure 9b shows initial crack deflection (*δ*_1_) remaining fairly constant with CFRP addition, while peak deflection (*δ*_p_) significantly increases, most notably at 19 mm displacement. The deflection at 80% post-peak load (*δ*_80%PPL_) also increased with CFRP displacement, indicating increased ductility. The extent of ductility enhancement is such that *δ*_p_ surpasses the L/150 serviceability limit for all CFRP-reinforced samples, resulting in negative *δ*_(δ_p_-L/150)_ values. A comparative analysis of deformation behavior reveals that unreinforced MPC specimens exhibit lower deflection capacity than CFRP-enhanced FMPCs, yet demonstrate greater deformation than fiber-modified MPC lacking CFRP reinforcement. This observation suggests CFRP incorporation may impart ductility enhancement effects even in the absence of discrete fiber reinforcement.

#### 3.2.3. Other Flexural Parameters

**Figure 10:**
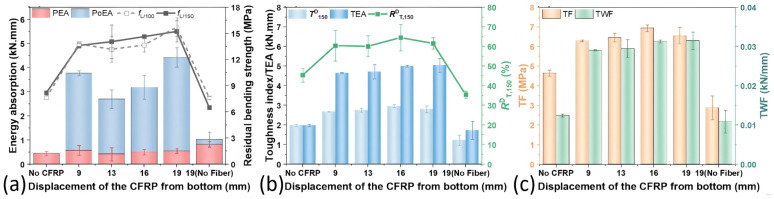
Flexural parameters (**a**) energy absorption (kN·mm) and residual bending strengths (MPa), (**b**) toughness index (kN·mm) and *R*^D^_T,150_, and (**c**) TF (MPa) and TWF (kN/mm) of optimal fiber MPC reinforced with CFRP fabric. (Note: Error bars represent standard deviation (SD)).

Figure 10 depicts different parameters related to the energy absorption and toughness of FMPC with varying CFRP fabric displacements. As shown in Figure 10a, the CFRP presence slightly enhanced the PEA while significantly boosting the PoEA of the FMPC samples, particularly at a 19 mm displacement, indicating improved energy absorption post-peak. Samples without CFRP or fibers display markedly lower PoEA, underscoring the synergistic effect of fibers and CFRP on energy absorption.

In Figure 10b, the toughness index (*T*^D^_150_), total energy absorption (TEA), and equivalent flexural strength ratio (*R*^D^_T,150_) increased with added CFRP, though CFRP displacement had minimal impact on these measurements. The low values in the no-fiber MPC reinforced with CFRP highlighted the crucial role of fibers in conjunction with CFRP to enhance the toughness of MPC materials. The toughness factor (TF) and the total work of fracture (TWF) also improved with the combined addition of fibers and CFRP (Figure 10c). However, these properties show little sensitivity to CFRP displacement, indicating that varying the placement of CFRP does not significantly affect toughness adjustment.

#### 3.2.4. Discussion

The synergy between fiber reinforcement and CFRP significantly enhances MPC’s mechanical properties, toughness, and energy absorption. Fibers provide internal support, improving crack resistance and stress distribution [58,59]. CFRP, with high tensile strength, augments load-bearing capabilities [60]. This cooperation increases material toughness, enabling greater energy absorption and crack resistance. Fibers bridge gaps, limiting crack widening and contributing to post-crack load-bearing [61]. Simultaneously, CFRP externally reinforces, restrains cracks, and adds bending strength [61].

Experimental results revealed that variations in CFRP placement had a marginal effect on fracture toughness, but substantially modified both ultimate flexural strength and cumulative energy absorption characteristics. Optimal CFRP placement leveraged flexibility and tensile properties over a larger deformation range. The experimental findings identify 19 mm as the optimal CFRP displacement, achieving peak mechanical performance and energy absorption while maintaining structural stability. This positioning balances reinforcement efficiency with material integrity, suggesting that precise CFRP placement and judicious fiber dosage selection are critical design parameters. Such optimization not only enhances load-bearing capacity under service conditions but also ensures cost-effective material utilization without sacrificing structural resilience.

### 3.3. Effect of the Displacement of CFRP Fabric

#### 3.3.1. Mechanical Strengths and L-D Curves

To enhance graphical clarity in the following sections, the specimen labeling has been simplified while maintaining all experimental parameters: the fiber content is fixed at 1.5 wt.% and CFRP reinforcement is consistently positioned at 19 mm displacement. The original designation T_19_-FMPC-V_1.5_ has been streamlined by omitting the CFRP position indicator T_19_, with specimens simply marked as either with or without CFRP reinforcement. Furthermore, while the fiber blend composition remains distinguished by type identifiers (1-4), the fiber dosage subscript (1.5) has been excluded from the figure labels to minimize visual complexity.

**Figure 11:**
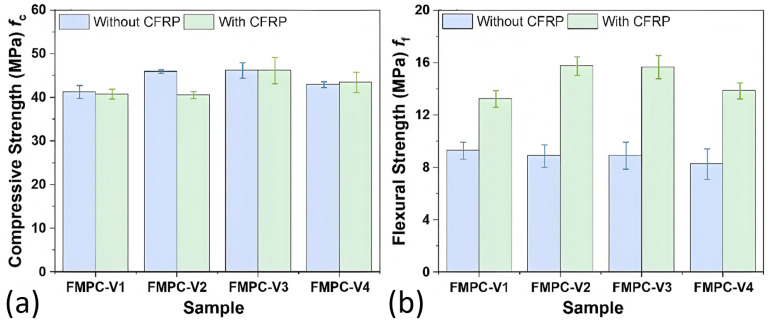

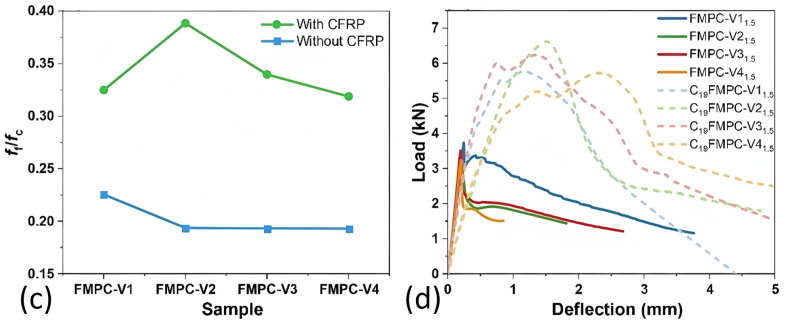
Properties (**a**) compressive strengths (MPa), (**b**) flexural strength (MPa), (**c**) the ratio of compressive strength to flexural strength (*f*_f_/*f*_c_) and (**d**) L-D curves of MPC-V with fibers and CFRP fabric. (Note: Error bars represent standard deviation (SD)).

Table 7 and Figure 11 illustrates the influence of CFRP fabric integration (positioned at 19 mm displacement) across various FMPC-V formulations, evaluating the universality of its synergistic reinforcement mechanism with discrete fibers. The comparative analysis demonstrates the consistent enhancement of mechanical properties, confirming the robust cooperative effect between CFRP and fiber reinforcement in MPC composites. The *f*_c_ and *f*_f_ results demonstrate that CFRP significantly boosted *f*_f_ but had minimal impact on *f*_c_, as shown in Figure 11a,b. The ratio *f*_f_/*f*_c_ in CFRP-enhanced FMPC-V is at least 1.5 times greater than in samples without CFRP, with the greatest gains observed in the specific fiber composition of CF/WSF/CPS_15_ = 1/1/1 (Figure 11c). CFRP also altered the flexural L-D curves, presenting a lower initial slope, higher peak load and deflection values, and a more gradual decline after the peak. These results underscore the significant role of CFRP in improving the flexural performance of FMPC- samples.

#### 3.3.2. Flexural Strengths, Moduli, and Deflections

**Figure 12:**
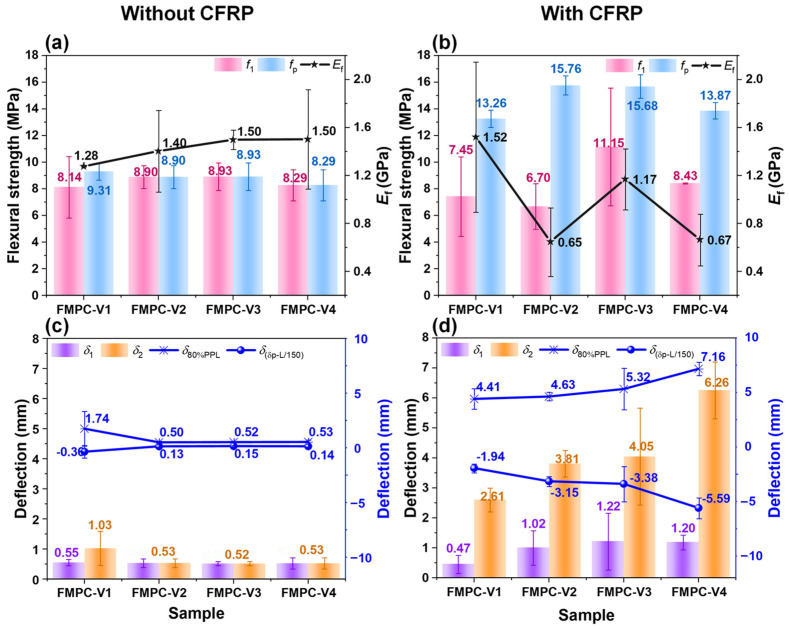
Flexural strength (MPa) of fiber-reinforced samples (**a**) with and (**b**) without CFRP fabric; deflection (mm) of fiber-reinforced samples (**c**) with and (**d**) without CFRP fabric. (Note: Error bars represent standard deviation (SD)).

Figure 12 details the effects of CFRP on the flexural performance and deformation characteristics of various FMPC-V samples. Without the CFRP addition, it is noted that the *f*_1_, *f*_p_ and *E*_f_ showed little variance among the samples (Figure 12a). Incorporating CFRP notably enhanced the *f*_p_ across all FMPC-V types, with a marked *f*_1_ increase in FMPC-V3 which contains a specific fiber blend of CF/WSF/WW = 1/1/1 (Figure 12b). In contrast to the flexural strengths, the *E*_f_ decreased slightly, which could be interpreted as an increase in flexibility or a decrease in stiffness due to the CFRP incorporation [62]. Deflection analyses (Figure 12c,d) reveal that CFRP significantly improves both the *δ*_1_ and δ_p_, showing enhanced ductility. The growth in *δ*_80%PPL_ was also noticed by the CFRP addition, and the shift in the *δ*_(δ_p_-L/150)_ to negative values occurred due to the significant δ_p_ enhancement. These changes suggest that the CFRP addition extended the deformation capacity of the FMPC-V samples before serviceability failure, potentially enhancing toughness and energy absorption [63].

#### 3.3.3. Other Flexural Parameters

**Figure 13:**
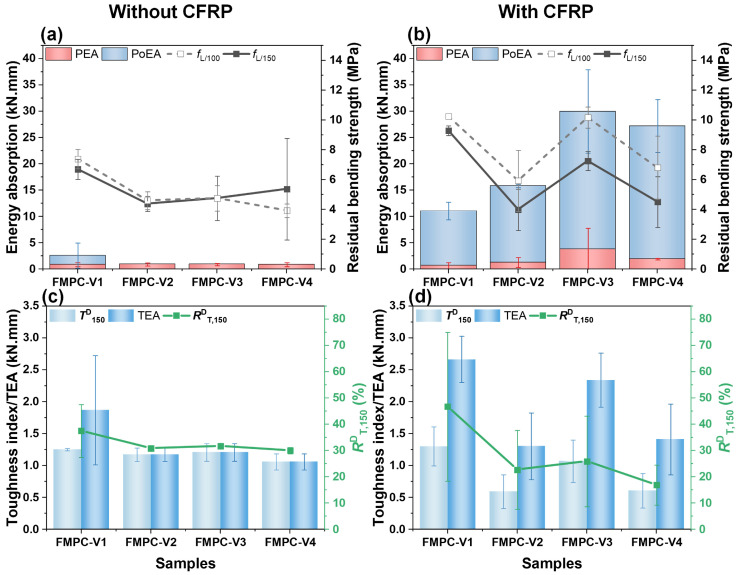
Energy absorption (kN·mm) and residual bending strengths (MPa) of fiber-reinforced samples (**a**) with and (**b**) without CFRP fabric; toughness index (kN·mm), and *R*^D^_T,150_ of fiber-reinforced samples (**c**) with and (**d**) without CFRP fabric. (Note: Error bars represent standard deviation (SD)).

A comparative evaluation of energy absorption capacity, residual flexural strength, and toughness indices between CFRP-reinforced and non-reinforced FMPC composites is presented in Figure 13. Without CFRP, the FMPC-V samples demonstrate consistent energy absorption and residual bending strengths (Figure 13a), indicating uniform performance across the board. The incorporation of CFRP reinforcement induces a significant enhancement in both peak energy absorption (PEA) and post-cracking energy absorption (PoEA), particularly for FMPC-V3 and FMPC-V4 composites (Figure 13a,b). This pronounced improvement demonstrates the material’s superior energy dissipation capacity following initial crack formation. However, compared to the PEA and PoEA, the increases in residual bending strength (*f*_L/100_ and *f*_L/150_) with CFRP were modest, suggesting only slight improvements in the load at the ultimate deformation.

Comparing the results in Figure 12c,d, the CFRP presence substantially boosted the TEA, especially for FMPC-V3, which underscores its role in augmenting overall energy absorption. Alterations in the *T*^D^_150_ and *R*^D^_T,150_ were minimal, with some FMPC variants (FMPC-V2 and FMPC-V4) even experiencing reductions, hinting at potential inconsistencies in sample performance.

**Figure 14:**
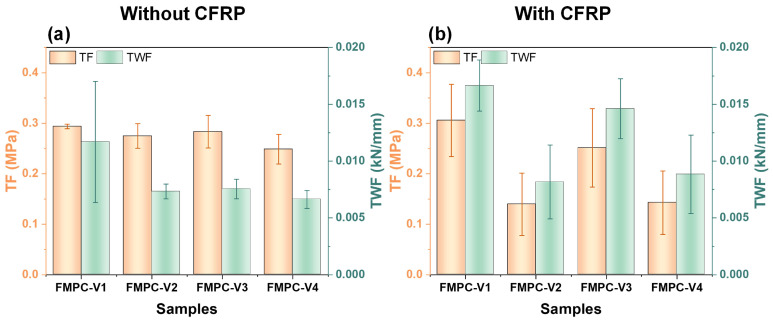
(**a**) Toughness factor (TF, MPa) and (**b**) total work of fracture (TWF, kN/mm) of Fiber-reinforced samples (**a**) with and (**b**) without CFRP fabric. (Note: Error bars represent standard deviation (SD)).

Figure 14 presents the TF and TWF of various FMPC-V samples with and without the CFRP addition. Without CFRP, TF and TWF are relatively uniform across the samples, with the error bars indicating minor variability (Figure 14a). The incorporation of CFRP reinforcement consistently enhances TWF, with FMPC-V3 exhibiting the most pronounced improvement among all tested formulations, demonstrating superior fracture resistance characteristics. However, TF did not consistently increase. FMPC-V2 and FMPC-V4 displayed a decrease, reflecting the variable impact of CFRP on the different FMPC-V compositions. The larger error bars for TF with CFRP, particularly for FMPC-V1, suggest that the influence of CFRP on toughness is not uniform and highly dependent on each FMPC-V samples’ specific properties.

#### 3.3.4. Efficacy Evaluation

The investigation of the various FMPC-V samples reveals that CFRP significantly impacted the mechanical properties, toughness, and energy absorption. The CFRP addition enhanced *f*_f_ and PoEA, with minimal effects on the *f*_c_, emphasizing its efficacy in scenarios dominated by bending forces. Meanwhile, the improved TF and TWF were noticed in some FMPC-V samples, indicating improved crack resistance and energy absorption during fracture. However, FMPC-V2 and FMPC-V4 showed a limited improvement or even reduction in TF values, highlighting the dependency of the CFRP on specific FMPC matrix designs. Considering the data reliability and overall performance enhancement, FMPC-V3 was found to be the most suitable design for the CFRP fabric reinforcement among all the investigated FMPC-V samples here.

Variability in CFRP-reinforced FMPC samples stems from factors like fiber distribution, interface quality, and manufacturing variability. Larger error bars, especially in specific variants, suggest achieving a consistent and beneficial response may be challenging. Although CFRP enhanced certain FMPC-V properties, its application must be carefully tailored to each formulation, considering potential drawbacks, to ensure optimal benefits in specific scenarios.

### 3.4. Further Discussion

Without the addition of CFRP fabric, it is found that fiber dosage has a more pronounced impact on the properties of FMPC samples than fiber type. This dosage dominance arises because insufficient fiber content fails to establish comprehensive crack-arresting networks [59,64], making matrix defects the primary performance driver. Higher fiber loadings, while more effective in modifying MPC properties, introduced greater performance variability due to fiber distribution heterogeneity and suboptimal fiber–matrix interfaces [50,51]. Although standard deviation values are provided to quantify this variability, it is important to acknowledge that achieving consistent dispersion and repeatability at elevated fiber contents (e.g., 5.0 wt.%) remains challenging due to increased risks of fiber agglomeration and non-uniform stress transfer. These factors generate stress concentrations and porosity, creating inconsistent load transfer mechanisms that complicate practical implementation. However, it should be noted that the interpretation of fiber agglomeration effects is primarily inferred from mechanical data variations and published studies [50,51,52,53,54,55,56,57], as direct microstructural evidence (e.g., from SEM analysis) was not included in the current study.

**Figure 15:**
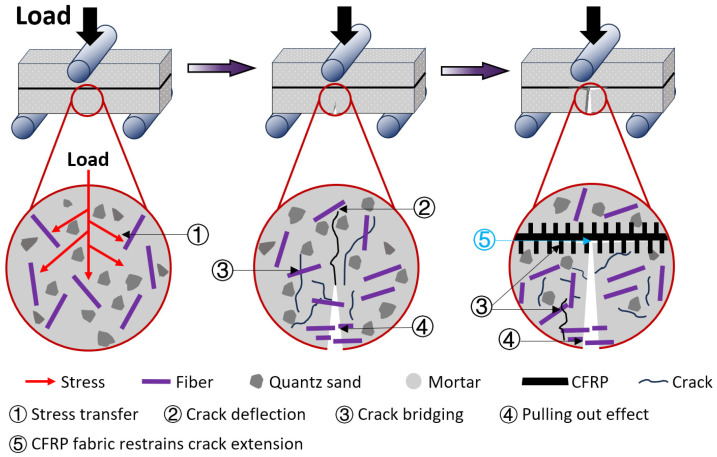
A schematic illustration of the combined effect of fibers and CFRP fabric, depicting the sequential activation of reinforcement mechanisms: pre-cracking stress transfer, microcrack deflection/bridging/fiber pull-out energy dissipation, and CFRP-mediated macrocrack restraint.

Introducing CFRP fabric to FMPC induces a synergistic interaction that leverages multi-scale toughening mechanisms, as illustrated in Figure 15. In the initial loading stage, fibers act as stress transfer mediators, redistributing localized stresses through interfacial bond development [65]. Upon initial cracking, fibers exhibit crack deflection, crack bridging, and pull-out effects, delaying the formation of critical macrocracks by dissipating energy through interfacial friction [66]. This microscale fiber reinforcement cooperates with macroscale CFRP confinement, where the high-tensile-strength CFRP fabric effectively restricts macrocrack propagation by interacting with fiber-mediated microcrack segmentation [67]. This synergistic mechanism activates fabric reinforcement prior to the formation of critical fracture planes, thereby simultaneously improving the flexural capacity and structural integrity of FMPC composites.

Additional cooperative effects extend beyond simple combined contributions. The embedded fibers create micro-roughness at the fabric–matrix interface, enhancing mechanical interlocking and bond strength beyond chemical adhesion limits. Fibers penetrating the CFRP–matrix interface induce radial compressive stresses, homogenizing strain fields and preventing premature interfacial decohesion. This multi-scale stress regulation ensures CFRP maintains consistent engagement throughout loading, leading to more uniform load distribution and superior composite performance.

The hybrid CFRP-fiber reinforcement system developed in this work demonstrates distinct advantages over conventional MPC composites, as evidenced by the comprehensive comparison in Table 8. Our system achieves an exceptional *f*_f_/*f*_c_ ratio of 0.389, substantially exceeding typical values of 0.107–0.228 reported for single-fiber MPC composites [14,25,43,68,69,70,71]. The exceptional *f*_f_/*f*_c_ ratio of 0.389 indicates a superior balance between flexural and compressive performance, effectively overcoming the common trade-off in traditional reinforcements. This enhanced balance is structurally vital, as it provides high resistance to bending and tensile stresses without significantly compromising compressive capacity, leading to improved durability and crack resistance under service loads, which is highly desirable in repair materials and structural components subject to dynamic or flexural loading. Notably, the embedded CFRP configuration outperforms externally wrapped systems [72] by providing more integrated mechanical enhancement while maintaining competitive compressive strength (~46 MPa) relative to natural fiber composites (~42–45 MPa) [70]. The results clearly establish that the strategic combination of hybrid fibers with internally incorporated CFRP fabric creates a more effective reinforcement architecture for MPC, delivering simultaneous improvements in both strength and toughness that surpass existing approaches. Furthermore, this hierarchical reinforcement strategy offers broader applicability to other brittle matrix systems, such as ultra-high-performance concrete, geopolymers, and ceramic composites, where balancing early-age strength and post-crack ductility remains a critical challenge. The design principles elucidated here, including fiber hybridization and internal fabric placement, can guide future developments in sustainable and resilient construction materials, particularly in applications requiring rapid repair and enhanced damage tolerance.

## 4. Conclusions

This study systematically investigated the cooperative reinforcement mechanisms of hybrid fibers and CFRP fabric in magnesium phosphate cement composites, yielding new scientific insights into multi-scale toughening strategies for brittle cementitious materials. The key scientific contributions are summarized as follows:

(1) The research establishes that fiber dosage exerts a more dominant influence on MPC properties than fiber type, with 3.5% fiber content providing the optimal balance between enhanced flexural performance (45% improvement) and minimal compressive strength reduction (<20%). Beyond this threshold, fiber agglomeration at 5.0% content introduces significant variability, highlighting the existence of critical fiber content limits for practical applications.
(2) A fundamental finding is the identification of 19 mm as the optimal CFRP fabric displacement, which maximizes the synergistic interaction between fiber reinforcement and fabric confinement. This specific configuration achieves an exceptional *f*_f_/*f*_c_ ratio of 0.389, demonstrating that strategic placement of internal CFRP fabric can overcome the traditional trade-off between compressive strength and flexural toughness in ceramic-based composites.
(3) The study further reveals that the cooperative reinforcement effect follows a hierarchical activation mechanism: microfibers initially bridge microcracks while CFRP fabric provides macro-scale crack restraint, with fiber–CFRP interfacial interactions enhancing stress redistribution. This multi-scale reinforcement strategy, validated across various fiber combinations, establishes a new paradigm for designing ductile MPC composites capable of transitioning from brittle to quasi-ductile fracture behavior.

These findings provide quantitative design guidelines for optimizing fiber–CFRP MPC systems and contribute fundamental knowledge to the broader field of composite material science, particularly for applications requiring rapid strength development and enhanced damage tolerance. However, it is also essential to recognize potential drawbacks such as the complexity of design, the potential for variability in manufacturing, and cost implications. Understanding these factors is vital for the optimization and practical application of fiber and fabric-reinforced MPC in construction and repair works. Future work should focus on long-term durability assessment, including 28-day mechanical performance and environmental resistance, along with microstructural characterization to further elucidate the interfacial mechanisms governing this hierarchical reinforcement system.

## Figures and Tables

**Table 1:** Physical characteristics of fibers used for producing specimens.

Name Abbreviation	Type	Length	Diameter	Color
CF	Cracked fiber	15 mm	18–48 μm	White
GF	Glass fiber	6 mm	13–15 μm	White
GFP	Glass fiber powder	-	150 μm	White
PMF	Polypropylene mesh fiber	19 mm	-	White
WSF	Waveform steel fiber	30 mm	0.2–0.6 mm	Silver
CPSF	Copper-plated steel fiber	10, 15 mm	0.18–0.25 mm	Brass
WW	Wave wire	11 mm	0.5–0.7 mm	Silver
SW	Straight wire	11 mm	0.5–0.7 mm	Silver

**Table 2:** Mixed designs for fiber MPC specimens.

Sample	MPP (g)	DBM (g)	BR (g)	QS (g)	W (g)	Fiber * (wt.%)	Fiber Type	CFRP Place **
MPC	459.5	540.5	54.1	500	250	0	-	-
FMPC	459.5	540.5	54.1	500	250	1.5 2.5 3.5 5.0	I-GF/CF = 1/1 II-GF/GFP/CF = 0.4/1.4/1 III-PMF/GF/CF = 0.4/1.4/1	-
C_n_FMPC-I_3.5_	459.5	540.5	54.1	500	250	3.5	I-GF/CF = 1/1	9 13 16 19
C_19_MPC	459.5	540.5	54.1	500	250	0	-	19
FMPC-V_1.5_	459.5	540.5	54.1	500	250	1.5	V1-CF/WSF/CPS10 = 1/1/1 V2-CF/WSF/CPS15 = 1/1/1 V3-CF/WSF/WW = 1/1/1 V4-CF/WSF/SW = 1/1/1	-
C_19_FMPC-V_1.5_	459.5	540.5	54.1	500	250	1.5	V1-CF/WSF/CPS10 = 1/1/1 V2-CF/WSF/CPS15 = 1/1/1 V3-CF/WSF/WW = 1/1/1 V4-CF/WSF/SW = 1/1/1	19

* Fiber (wt.%) indicates the fiber content of the MPC paste. ** CFRP place refers to the precisely controlled vertical distance (mm) from the specimen’s bottom reference plane to the centroid of the CFRP fabric.

**Table 3:** Flexural parameters and properties obtained from the L-D curves.

Symbol	Parameter	Definition/Equation	
*P*_1_	First peak load	Load causes the first cracking in the samples	
*P*_p_	Peak load	The highest load on the L-D curve	
*δ*_1_	Deflection at *P*_1_	Deflection at the *P*_1_ on the L-D curve	
*δ*_p_	Deflection at *P*_p_	Deflection at the *P*_p_ on the L-D curve	
*f*_1_	First peak strength	Strength calculated by (2), where *P* = *P*_1_	
*f_p_*	Peak strength	Strength calculated by (2), where *P* = *P*_p_	
80%PPL	80% of peak load	Load equals 80% × *P*_p_	
*δ*_80%PPL_	Deflection at 80% *P*_p_ in post-peak	80%PPL deflection on the descending curve after *P*_p_	
*δ*_δp-L/150_	Difference between *δ*_p_ and L/150	*δ*_δp-L_/150 = (L/150)-*δ*_p_ (L = 100 mm here)	(5)
*P*_L/150_	Load at L/150 deflection	Residual load at the deflection of L/150	
*f*_L/150_	Strength at L/150 deflection	Strength calculated by (2), where *P* = *P*_L/150_	
*T* ^D^_150_	Toughness index at L/150	The area under the L-D curve from 0 to L/150	
*P*_L/100_	Load at L/100 deflection	Residual load at the deflection of L/100	
*f*_L/100_	Strength at L/100 deflection	Strength calculated by (2), where *P* = *P*_L/100_	
*T* ^D^_100_	Toughness index at L/100	The area under the L-D curve from 0 to L/100	
*R* ^D^_T,150_	Equivalent flexural strength ratio (EFSR, implying flexural ductility)	$R_{T,150}^{D}\left(\%\right)=100\times \frac{150T_{150}^{D}}{f_{1}bd^{2}}$	(6)
TF	Toughness factor	$TF\left(MPa\right)=\frac{150T_{150}^{D}}{f_{1}bd^{2}}\times f_{1}=R_{T,150}^{D}\times f_{1}$	(7)
PEA	EA from 0 to *δ*_1_	The area under the L-D curve from 0 to *δ*_1_	
PoEA	EA from *δ*_1_ to *δ*_p_	The area under the L-D curve from *δ*_1_ to *δ_p_*	
TEA	Total EA	*T*^D^_150_ for softening deflection L-D curves *T*^D^_100_ for hardening deflection L-D curves	
TWF	Total work of fracture	$TWF=\frac{TEA}{bd}$	(8)
*E_f_*	Modulus of elasticity	$E_{f}=\frac{L^{3}m}{4bd^{3}}\;$	(9)

Notes: in this table, L is the span between the two bottom supports, equal to 100 mm; *b* is the width of the beam specimens, mm; *d* is the height of the beam specimens, mm; *m* is the slope of the L-D curve from 50 millionths to 40%*P*_p_, N/mm.

**Table 4:** Mechanical strengths of MPC reinforced with various types and dosages of fibers.

	Compressive Strength (CS)			Flexural Strength (FS)			FS to CS Ratios	
	*f*_c_ (MPa)	COV (%)	R (%)	*f*_f_ (MPa)	COV (%)	R (%)	*f*_f_/*f*_c_	R (%)
MPC	72.17	4.00	0.00	8.45	2.20	0.00	0.12	0.00
FMPC-I_1.5_	56.40	5.63	−21.85	6.02	9.80	−28.75	0.11	−8.83
FMPC-I_2.5_	55.00	2.41	−23.79	7.65	5.39	−9.39	0.14	18.89
FMPC-I_3.5_	60.53	0.25	−16.12	10.28	10.79	21.66	0.17	45.04
FMPC-I_5.0_	63.87	1.15	−11.50	10.96	4.40	29.70	0.17	46.56
FMPC-II_1.5_	53.17	2.08	−26.33	8.01	12.89	−5.16	0.15	28.73
FMPC-II_2.5_	64.53	0.36	−10.58	8.90	5.00	5.36	0.14	17.82
FMPC-II_3.5_	66.77	1.10	−7.48	10.77	5.00	27.55	0.16	37.87
FMPC-II_5.0_	58.10	1.34	−19.49	10.25	12.25	21.37	0.18	50.75
FMPC-III_1.5_	54.20	1.51	−24.90	9.67	1.78	14.48	0.18	52.43
FMPC-III_2.5_	50.70	0.90	−29.75	9.40	2.19	11.32	0.19	58.45
FMPC-III_3.5_	64.30	1.02	−10.90	9.47	5.11	12.10	0.15	25.81
FMPC-III_5.0_	59.67	2.58	−17.32	13.55	4.88	60.42	0.23	94.03

**Table 5:** *f*_c_, *f*_f_ and *E*_f_ of MPC reinforced with CFRP in different displacements.

CFRP Displacement (mm)	*f*_c_ (MPa)	SD	*f*_f_ (MPa)	SD	*E*_f_ (GPa)	SD
9	55.90	±0.78	14.02	±0.27	1.56	±0.10
13	54.43	±3.87	14.10	±1.40	1.52	±0.28
16	57.87	±2.16	14.76	±0.29	1.78	±0.25
19	50.27	±0.59	15.88	±1.28	1.41	±0.11

**Table 6:** Toughness index (TEA and *T*^D^_150_), TF and TWF of MPC reinforced with CFRP in different displacements.

CFRP Displacement (mm)	Toughness Index (kN·mm)				TF (MPa)	SD	TWF (kN/mm)	SD
	*T* ^D^_150_	SD	TEA	SD				
9	2.68	±0.02	4.64	±0.03	6.28	±0.04	0.029	±1.96 × 10^−4^
13	2.75	±0.09	4.72	±0.37	6.45	±0.22	0.029	±2.30 × 10^−3^
16	2.96	±0.07	5.00	±0.06	6.94	±0.17	0.031	±3.45 × 10^−4^
19	2.80	±0.18	5.04	±0.36	6.56	±0.42	0.032	±2.23 × 10^−3^

**Table 7:** Mechanical strengths of MPC reinforced with various fiber combinations and CFRP.

	With CFRP				Without CFRP				With CFRP	Without CFRP
	*f*_c_ (MPa)	SD	*f*_f_ (MPa)	SD	*f*_c_ (MPa)	SD	*f*_f_ (MPa)	SD	*f*_f_/*f*_c_	
FMPC-V1	40.80	±1.49	13.85	±1.13	41.30	±1.14	8.77	±1.04	0.34	0.21
FMPC-V2	40.57	±0.40	15.76	±0.72	45.97	±0.81	8.90	±0.86	0.39	0.19
FMPC-V3	46.17	±1.76	15.68	±0.88	46.23	±3.03	8.93	±1.03	0.34	0.19
FMPC-V4	43.50	±0.68	13.87	±0.61	42.97	±2.34	8.29	±1.16	0.32	0.19

**Table 8:** Summary of research results on properties of fiber-reinforced and CFRP-reinforced MPC.

References	Fiber Characteristics		CFRP	*f*_c_ (MPa)	*f*_f_ (MPa)	*f*_f_/*f*_c_
	Type	Content				
This work	CF/WSF/CPS15	1.5 wt.% (1:1:1)	Embedded	40.57	15.76	0.389
Dong [25]	PVA	0.5 wt.%	None	57.8	10.3	0.178
Ahmad [68]	BS	0.5 vol.%	None	49.4	5.3	0.107
Feng [69]	PVA + MS	1.6 vol.% + 0.6 vol.%	None	53.5	10.6	0.198
Qin [14]	BS	1 vol.%	None	77.6 (28 d)	N/R	N/R
Qin [14]	BS	1.5 vol.%	None	N/R	5.06 (7 d)	N/R
Liu [70]	BC	10 wt.%	None	44.5	9.3	0.208
Liu [70]	BC	20 wt.%	None	41.6	9.5	0.228
Zhang [71]	Sisal	30%	None	80	N/R	N/R
Zhang [43]	BS	1.2 vol.%	None	ca. 60 (3 h)	ca. 12.5 (3 h)	ca. 0.208
Zhang [43]	BS	1.2 vol.%	None	ca. 88 (3 h)	ca. 14 (3 h)	ca. 0.159
Zhang [72]	N/R	N/R	Wrapped	+47.1%	N/R	N/R
Feng [62]	N/R	N/R	Wrapped	N/R	+370%	N/R

Note: PVA = Polyvinyl alcohol; BS = Basalt; MS = Micro steel; BC = Brucite; N/R indicates that the cited reference did not report the corresponding property.

## Data Availability

The data presented in this study are available on request from the corresponding author.
